# Freedom and need: The evolution of public strategy for biomedical and health research in England

**DOI:** 10.1186/1478-4505-6-2

**Published:** 2008-01-29

**Authors:** Miriam Shergold, Jonathan Grant

**Affiliations:** 1 RAND Europe, Westbrook Centre, Milton Road, Cambridge CB4 1YG, UK

## Abstract

The optimal support of health-related research and development with public money is a complex challenge. Over the last century, policy makers in England have conceived and implemented a variety of models, ranging from independent, curiosity driven research to needs-based state commissions, and promoting different bodies to oversee scientific work. This paper traces these approaches, identifies the principles that drove them, and discusses their role in shaping policy for publicly funded health research, up to the recent launch of a new research strategy by the Department of Health.

## Introduction

Ever since the emergence of the UK's first public funds for biomedical research in the early 20^th ^century, policy makers have sought ways of optimising returns on this investment in the near and long term. It is a pertinent challenge, as the price of poor decisions is paid in the currency of human suffering. Science support is always vulnerable to practical pitfalls, such as budget cuts and capacity shortages. At the same time, however, policy makers face questions of principle, such as the appropriate balance between curiosity-driven and needs-driven research, and the appropriate degree of direction by the state. Further choices are to be made with regard to priorities, and the individuals best placed to set them.

In England, the debate of these issues stretches back to the first emergence of public funds for health research. This essay traces the research strategies through which the Department of Health, and its partners, have endeavoured to enhance national well-being. The rationale for the paper was to put into context the development and formulation of a research and development strategy for the Department. On this basis, the paper is also an interesting example of using historical accounts to understand past policy change, with the aim of informing current and future policy making. The paper discusses the responses of different generations of policy makers to the perennial questions of public support of biomedical research, from the beginning of the 20th century to the most recent reform plans.

## The beginnings (1911 to 1946)

The first public fund for health-related research for the benefit of the British population was established through the National Insurance Act 1911. Recognising the need for systematic support, the government pledged that for every individual insured, one penny of public funds would be set aside in the newly created Medical Research Fund. The sum generated in this way was considerable, amounting to an estimated £40,000 to £60,000 per year [1,2].

In 1913, a Medical Research Committee was appointed to oversee the research activities supported by the new fund, the scope of which was simply defined as 'medical research' [3]. The committee comprised representatives from the House of Lords and House of Commons, as well as six scientific members, 'men of eminence in the most important departments with which the Committee is concerned, namely, pathology, bacteriology, medicine, surgery, bio-chemistry and public health' [2].

In the early years of the Committee, no Ministry of Health existed. Responsibility for health services was divided between various government bodies, such as the Education Board and the Home Office. 'Researches into medical questions', on the other hand, were spread across the Admiralty, War Office, Ministry of Munitions, Local Government Board, and Board of Control [2]. This fragmented situation is likely to have enhanced the freedom of the Medical Research Committee's researchers, whose programme of work was approved by the commission administering the National Health Insurance. The Committee were keenly aware that this freedom was a privilege, and that state support often came at the price of state control. In 1915, its members praised their supervising body for having allowed them 'the most complete freedom... to bring flexible and rapid assistance to the national need on occasion of emergency with the least possible delay in the motion of the constitutional machinery' [2,4].

At the end of the First World War, the government made plans to create a Ministry of Health, a new central body with scope for unprecedented co-ordination of health-related concerns. Given that health research could be seen to fall into this remit, the future relationship of the Medical Research Committee and the new Ministry needed to be clarified. Influential writings by Lord Haldane, the former Lord Chancellor, and by Christopher Addison, chairman of the Reconstruction Committee discussing the proposals for the new Ministry, defended the principle of independent and undirected research.

Lord Haldane's 'Report of the Machinery of Government Committee', published in 1918, conceived the Ministry and the Medical Research Committee as two separate, but communicating, bodies concerned with different kinds of research. The Committee was to continue independent, opportunity-led research with potential relevance to various government departments ('general use research') [2]. Government departments, on the other hand, were encouraged to undertake research to inform administrative decisions ('operational research', such as surveys), rather than influence or rival the scientific activities of the independent body [2]. To justify this division, Haldane pointed out that by tradition, the work of the Committee had 'never been confined to the investigation of questions suggested by the current administration of the Health Insurance Acts'.

Addison re-iterated Haldane's arguments for independent research in a memorandum published in 1919. To demonstrate the success of the 'general use' approach, he pointed out that research into oxygen had resulted in advances in aviation, mine rescue, and pneumonia treatment. As such, the work had been of relevance to several different government departments [5]. Convinced that only scientific freedom could produce the highest quality work, Addison worried that departmental direction would force scientists to concentrate too much on immediate needs. Going further, he warned that ministerial control would also jeopardise the rigour scientific research, as 'a keen and energetic minister... would constantly be tempted to endeavour in various ways to secure that the conclusions reached by organised work under any scientific body... should not suggest that his administrative policy might require alteration' [6].

Addison's plea to keep the Committee scientists aloof from current demands created a research vacuum for the new Ministry to fill. Whilst Haldane's vision of Ministry research had been largely limited to surveys and statistics, Addison envisaged a degree of parity:

*The question is *how much* medical research can best be carried out by a medical staff in close relation to the administrative side of the Ministry, and how much can best be carried out by a body whose work will be less immediately directed towards the current administration of health matters.*

The memorandum concluded that due to its natural bias towards pressing problems, Ministry research should respond to immediate needs, whereas Committee work should remain research-driven [6]. It thus guarded the freedom of Committee researchers, but at the same time launched the Ministry as a biomedical research body in its own right. Sir Walter Morley Fletcher, secretary of the MRC, promoted a rather different scenario: not only must the organisation be shielded from external control, but it should act as national body directing all medical research, including work funded through private donations [7].

The following official arrangements largely reflected Addison's proposals. The new Ministry was created, and the Medical Research Committee remained as a separate and independent body, now called the Medical Research Council (MRC). However, relations between the two bodies grew increasingly tense, especially when a Departmental Cancer Committee was set up by the Ministry in 1923 [7]. To resolve the situation, in the following year, Sir Walter Fletcher and Sir George Newman, the Ministry's Chief Medical Officer, privately drew up a concordat which confirmed each body's profile [7,8]. The young Ministry was to 'provide investigation... of scientific problems arising in the current administrative work of the Ministry', and undertake 'research by such investigations as can best be carried out by the Ministry, in the interests of public health administration, applied knowledge or medical services'[9].

Although its remit was now clearly mapped out, government-led research was slow to gain momentum due to inadequate external structures and internal capacity. For example, in 1932, the Ministry created a Committee on Immunisation with a strong research component, but subsequent inertia left local authorities in charge if immunisations [10]. In theory, the Ministry was uniquely placed to combine health research with health care. In practice, health care provision was regionally and socially fragmented, making it difficult for the Ministry to embrace its new identity as leader in applied research. This situation changed fundamentally in 1946, when the National Health Service (NHS) was created. In a radical departure, the NHS offered a unified system for healthcare provision across the nation, open to everyone and free at the point of delivery. The Act explicitly put the Minister of Health in charge of 'research into matters relating to the causation, prevention, diagnosis of illness or mental defectiveness', a broad remit reminiscent of the original brief of the Medical Research Committee [11,12]. However, vision was not matched by means, and although clinical research began to expand in teaching hospitals, the Ministry's actual research programme remained confined to public health [13]. Consequently, existing structures prevailed until the 1960s, when external pressures re-fuelled interest in the Ministry as a significant player in the research arena.

## Struggle over territory (1964 to 1971)

The period following the National Health Service Act was characterised by a growing awareness of science as a national priority, as well as changes in the way that science was undertaken [1]. This development upset the carefully negotiated balance of responsibilities between the MRC and the Ministry. Large-scale medical advances, such as population screening and organ transplantation, required structures beyond those which the MRC could offer. The Ministry stepped in to fill the gap by developing its own research capacity and research units. At the same time, the MRC engaged in public health work alongside the Ministry. The line between the research territories of Ministry and Council thus could no longer be drawn along the traditional idea of 'immediate' or 'applied' and 'general' work. Overlapping concerns and resulting attempts by the Ministry to influence the MRC agenda resulted in frictions, for example, when the Chief Medical Officer observed a lack of epidemiological focus in the Council's clinical work. Matters were not helped by the fact that some in the community still regarded ministry researchers as 'second-class scientific citizens' [14]. Wider society, meanwhile, began to exhibit signs of disenchantment with science, in some parts amounting to a veritable science counter culture [15].

By the 1960s, the blurred relationship between the MRC and the Ministry (from 1966, the Department of Health and Social Security, DHSS) reflected a wider debate in government about the best way to undertake publicly funded research. In 1964, a government-commissioned inquiry into civil science led by Sir Burke Trend found that endeavours had been weakened by a lack of clarity in 'the arrangements for co-ordinating Government's scientific effort and for apportioning the available resources between agencies on a rational basis' [16].

The need for a more organised approach gave rise to the idea that government departments should set the agenda for all publicly funded research in their field. In the case of health research, this challenge to the MRC had not been seriously considered since Haldane and Addison had defended its independence in 1918/19. In 1970, the idea of bringing research councils under departmental control was spelled out in an unpublished report on the Agricultural Research Council led by Paul Osmond of the Civil Service Department [8,14]. Shortly afterwards, a new government under Edward Heath was elected, but the drive towards reform in the management of civil science continued. To inform their own future strategy, the new government commissioned two reports, which were published as appendices to a Green Paper in 1971.

The first report, written by Lord Rothschild, head of the Central Policy Review Staff, examined the department and council system with the aim of determining 'the most effective arrangements for organising and supporting pure and applied scientific research and post-graduate training'. Rothschild believed that the individualistic stance exemplified by the MRC was to blame for the perceived unsatisfactory return on public investment in research. Effective service for the government, he argued, required a centralised and needs-focused approach. Administrative departments, he observed, did not require 'scientific support' but 'applied R&D, to achieve specific predetermined objectives' [17]. Rothschild's solution was a radically new approach based on precise departmental commissions: 'Applied R&D... must be done on a customer contractor basis. The customer says what he wants; the contractor does it (if he can); and the customer pays' [17].

The report envisaged the government departments as the customer, or acting on behalf of the ultimate customers, and the research bodies as the contractor. To empower the customer, the report proposed that a large part of the funds previously allocated to the research councils should be transferred to the departments. The Research Councils would then have to win back this money by bidding for departmental research contracts. At the same time, they would not normally be able to refuse commissions by the departments. Curiosity-driven research, the traditional domain of the councils, was to be financed by a 'general research surcharge' factored into the price of commissioned work. This system, Rothschild expected, would end the lingering 'scientific snobbery' dividing 'the 'haves' in the Research Councils and the 'have nots' in the Departments'. After all, government department dissatisfied with council work would 'go elsewhere, with their money, to get their objectives met' [17].

Rothschild negated Haldane's and Addison's endorsement of research council independence. He equally rejected that departments and councils should occupy different positions on the basic-applied research spectrum [1,17]. A third radical postulate called for the national research agenda to be taken out of the hands of scientists, for 'however distinguished, intelligent and practical scientists may be, they cannot be so well qualified to decide what the needs of the nation are, and their priorities, as those responsible for ensuring that those needs are met' [17]. However, Rothschild did not propose a department-led national strategy for health-related research and development. Priority setting, for him, was a question of identifying immediate needs. Accordingly, he felt no need to track national R&D activities, arguing that such 'general oversight would serve no useful purpose' [17].

The second report published with the Green Paper, 'The Future of the Research Council System', scrutinised Osmond's proposal to bring the research councils under ministerial control. Led by the distinguished academic Sir Frederick Dainton, it concluded in favour of the councils' autonomous status. Echoing Addison's thoughts on the relationship between government and science, Dainton highlighted that independent research remained of great value in public decision making: 'Departments... need to be able to obtain help and independent advice... It is essential that the advice and information from this source should be free from considerations of administrative and political convenience' [18].

Unlike Haldane and Addison, Dainton thus no longer celebrated council independence as good in itself with long-term benefits for the nation. Instead, the report stressed the utility of unbiased expertise for the functioning of the departments. In this way, both Green Paper reports conceptually moved the departments centre stage.

The Research Councils emerged bowed, but not broken from the first major challenge to their status. A White Paper published in July 1972 ensured their survival, but implemented most of Rothschild's proposals with the addition of the supervisory Board of Research Councils recommended by Dainton. In the area of health, this shift catapulted the Department of Health and Social Security (DHSS) into a national management and leadership position. As the paper stated, 'final responsibility for defining the objectives of commissioned work must rest with the Department concerned' [19].

However, the Department's research management experience was still limited. As the White Paper itself had observed, the Select Committee on Science and Technology had found not long before that 'at present, neither Parliament nor the public is given sufficient information about departmental research and development programmes'. The Department's research and development budget for 1972–73 amounted to £13 million, including £9.3 million for current expenditure. Of the latter sum, only £0.7 million were earmarked for intramural work [19].

The government appointed a Chief Scientist to oversee the Department's expanded research and development programme. The Chief Scientist was supported by a small body of staff and several advisory bodies. Most notably, the Chief Scientist's Research Committee discussed suitable foundations for commissioning, such as cost-benefit-analysis and the analysis of future needs [8,19,20]. At the same time, £5 million out of the £20 million of public funds for the MRC were transferred to the Department. The sum represented the estimated 25% of MRC funding previously spent on 'applied' research [14,19]. In addition, the Department was now represented on the Board of the MRC as proposed by Rothschild.

The reforms allowed for stronger central direction, but were predictably unpopular with the science community. In particular, researchers protested that the government had not properly analysed the supposed weaknesses of the previous system [1]. As the reforms were implemented, practical problems emerged. For example, the research councils found it difficult to raise funds for non-commissioned, capacity-building research, because government departments were under no obligation to pay the new 'general research surcharge' [21]. Moreover, researchers were disappointed that science had not gained more influence on policy making [1].

The subsequent relationship between the DHSS and MRC revealed that departmental structures were insufficiently robust to allow authoritative decision-making within the new system. The DHSS not only needed to handle a greatly expanded budget, but was also expected to allocate funding across all areas of health research. Given the limited capacity of the Chief Scientist's office, this proved an overwhelming challenge. The problem was resolved by giving the MRC broad research contracts, which effectively allowed the Council to continue its existing research programme. This move was pragmatic, but ignored Rothschild's outspoken opposition to broad or open-ended funding agreements. MRC researchers, meanwhile, continued to feel the reforms as a heavy administrative burden, compounded by time consuming obligations to act as expert advisers to the government [14,15].

The first radical re-structuring of the system for public health had put customer and customer need at the centre of funding decisions. However, reality fell short of vision because neither customers nor needs were adequately represented by the DHSS. Nevertheless, the Department had been promoted to a leading strategic role, and from henceforth was a research and development player to be reckoned with.

## Reform and re-definition (1975 to 1981)

The disparity between the government's aspirations for the new system and its practical shortcomings did not go unnoticed, leading to a series of reports and subsequent reforms. In 1975, a report by Maurice Kogan on the Department's research activity highlighted how arrangements differed from what had been envisaged by Rothschild. The Chief Scientist's organisation was on the margins rather than at the centre of the organisation. Worse, a severe lack of scientific experience among staff hampered the Department in devising its own projects:

*From the medical science viewpoint it was thought that the Department was lacking staff who had sufficient research experience, who were neither researchers nor practitioners, but administrators and overall the Department was not sufficiently strong scientifically to generate its own projects*[14,22].

In response to the report, the internal structures were re-modelled. The result was a Chief Scientist's Research Committee informed by more than ten Research Liaison Groups (RLGs), which brought together departmental advisers, independent experts, and DHSS staff [8]. Greeted by some as the most successful attempt at establishing the customer/contractor principle, the new construct was soon criticised for its lay members and an overambitious range of subjects to be covered by each group [14].

Around the time of the formation of the RLGs, in 1978, a report by a working party of the Nuffield Provincial Hospital Trust criticised the Department for lacking 'a definable research policy', and sufficient overview of the fragmented, customer-focused R&D activities nominally under its control [13,14]. In the following year, a review of civil service management in the DHSS also diagnosed shortcomings in arrangements for research and development [8].

The government's own review of the Rothschild reforms in 1979 made an altogether more positive assessment. However, it acknowledged that the continuation of the MRC programme under broad *pro forma *commissions contravened the customer-contractor principle [1]. The review traced this problem to strict constraints on administrative and staff costs, due to which the Department had 'not felt justified in developing expertise for a full commissioning role in the biomedical area' [23]. In response, departmental funds for health and social security research were put under the direct control of the Chief Scientist. At the same time, a new concordat provided that the MRC would officially regain its funds, but liaise closely with the Department in shaping its research programme.

The outcome of the reforms, reports and debates of the 1970s was a hybrid of pre- and post Rothschild models. The bid to create a single, needs-focused decision making body had been unsuccessful, leaving the Department and the MRC to co-exist as largely separate research bodies. This raised once more the question of the appropriate balance between independent and commissioned, curiosity-led and needs-based research and development. Other issues, such as the adequate range of science to support, and an effective linking up of science and policy, also remained to be solved in the inevitable next round of improvement efforts [20].

## The rise of NHS Research and Development (1988 to 1993)

As the new decade dawned, a feeling of crisis in national science intensified the ongoing debate about the direction and support of research by the state [24]. The previous years' exponential growth in the government's civil science came to a halt, leading to cuts in departmental programmes [25]. At the same time, a fall in the UK's share of publications and citations suggested declining scientific standards [26,27]. As the government now considered applied or 'near market' research as the responsibility of industry rather than government departments, there was also concern that national needs had grown to play a subordinate role in the public research agenda [25]. In the health sector, these developments had translated into worsening conditions for the conduct and dissemination of clinical research, which, in turn, affected the quality of patient care [27].

The need to contain health care costs fuelled the demand for change in the management of medical research. In 1988, the report 'Priorities in Medical Research' by the House of Lords Science and Technology Committee reviewed the organisation of medical research in the UK and within the DHSS [24,27]. By this time, health-related research was taking place under the auspices of three principal bodies: the MRC, the Department of Health, and the NHS. The MRC carried out both basic and clinical science, but in line with the new concordat had also started to support some health services research. The Centrally Commissioned Programme of the Department of Health focused on health services research and public health research, with the aim of providing evidence for government policy making. Finally, the NHS had also begun to sponsor a modest amount of research across the disciplines. There had been attempts to co-ordinate all of these activities and to link them with those of the medical research charities, but no thorough success had been achieved [27].

The Committee report approached the problem by re-visiting the fundamentals of public science support. It discussed in unprecedented detail the potential and limits of targeted research, presented stakeholder opinions on the optimal balance of basic and applied research, and cited various mechanisms for priority setting, for example, research spending proportional to overall spending in a particular area or discipline, or proportional to the number of people affected. The Committee did not take a clear position on priorities but proposed that overall, research should be led by science rather than problems:

Better results will be achieved by supporting good ideas and advances in science as they arise, than by concentrating on recognised problems regardless of whether promising leads are in prospect... the main focus of public policy in medical research should be the establishment of a strong infrastructure for research in well-found laboratories and the supply of a strongly motivated and trained research community.

Scientists' influence in shaping their own research activities thus was rehabilitated. To reinforce the point, the committee also concluded that 'the Chief Scientist's Office in the DHSS may be adequate for the Department's internal purposes but it has certainly not proved capable of supplying the informed customer for health care envisaged by Lord Rothschild' [24].

However, these observations did not mean that all faith in a centrally run applied research programme was lost. Instead, the Committee promoted the NHS to fill the role by operating its own, distinct and formally recognised R&D programme. After decades of rivalry between Department and the MRC, a new contender had quietly emerged. The Committee envisaged that in the new medical *trias*, the Department would return to a more limited research role, reminiscent of the responsibilities once conceived by Haldane:

*The DHSS and the NHS both require research programmes but these will be different in scale and kind. There is a clear distinction between the needs of ministerial policy and NHS research*[24].

The proposals were generally well received, and led to the creation, in 1991, of a NHS R&D programme with a single management structure and an integrated system of decision-making, research, delivery, and management based on a regional substructure. The Director of the new programme had responsibility for both NHS R&D and for the Department's Centrally Commissioned Programme [12,27].

In his strategy for the new R&D programme, Sir Michael Peckham, the newly appointed Director, stressed the aims of coherence and effective translation of activities within the wider programme of the Department, by now called the Department of Health (DH). Priorities were to be set by the Director with advice from a Central R&D Committee (CRDC) informed by stakeholders including patient groups and other research funders. Areas of research of national relevance would be funded from a central budget, other relevant research would become the responsibility of the NHS regions. The strategy paper also defined the principal criteria for priority setting, including the burden of disease, prevalence, policy priorities, feasibility of research and potential benefits [13,28].

The first national commissioned research programme (1992–95) promoted data collection as the basis of future priority setting. Accordingly, its first project was a national stock-taking exercise, the 'Health Service Survey for 1991'. A following strategy paper furthermore proposed a systematic use of evaluations [29,30]. The same paper also observed a predominance of science-push in the research agenda, with 'insufficient attention being paid to a wide range of issues germane to health sector demands'[27]. The medical community expressed their own sense of imbalance. In particular, there was concern that despite its outward dedication to applied research, the new system lacked adequate incentives for clinical research [29].

In just half a decade, very significant changes had been put into train, and the NHS had been successfully promoted as the new arena of publicly funded, applied research. The move integrated departmental direction and frontline service delivery and opened up great potential for clinical investigation, although subsequently there was some criticism of evidence being selected to bolster ministers' perceptions [12].

To allow the NHS to achieve the desired *gravitas *as 'the' body for applied research, more than strategy was required. Ultimately, its success would depend on adequate structures linking departmental priorities with researcher activity, demand with capacity, and capacity with funding. All of these challenges were yet to be resolved.

## Reform of funding for NHS R&D (1994 to 1999)

Plans for clinical research under the auspices of the R&D directorate were visionary, but at the front line, conditions were discouraging. The NHS and Community Care Act (1990) had introduced an internal market system in which general practice fund holders and health authorities bought services from hospital trusts. In the resulting climate of cost awareness and competition, it had become relatively unattractive for Trusts to host research. By 1994, a report by the UK Coordinating Committee on Cancer Research described how market pressures had led health authorities and hospitals to cut costs and shorten inpatient times, leading to disruptions in research [31,32]. Scientific endeavour within the NHS, whilst having unique opportunities, thus was also highly vulnerable to the effects of internal management decisions.

To improve conditions, the Director R&D asked a task force led by the economist Professor Anthony Culyer to review existing arrangements for R&D in the NHS [33]. On the strategy level, the resulting report, 'Supporting Research in the NHS' (1994), concluded that greater coherence in activities had been achieved, but linkage with other research funders still left to be desired. Regarding the relationship of NHS and MRC, the report criticised the seemingly unlimited claim of the MRC on NHS service support in carrying out its own research, and called for a new concordat.

Just as Culyer and his team continued the tradition of reflecting on the appropriate relationship of the different national research bodies, they, too, needed to arbitrate between problems of immediate relevance, and longer-term endeavours. The task force took no defined position on this matter, but stated that the majority of its informants had opted for a combination, 'with more emphasis on research into the effectiveness and cost-effectiveness of health service provision'. With regard to priority setting, the report called for the consultation of more stakeholders, including purchasers and providers. It also advocated an enhanced regional dimension in determining needs and commissioning research.

Turning to the problem of costs, the task force proposed the creation of a single funding stream, to be partly financed by a levy on the budgets of all health care purchasers. In a departure from previous, mostly informal funding arrangements, it was proposed that in future, institutions would distinguish between the costs of healthcare, R&D and training, and receive dedicated research grants and budgets [27,33].

Cuyler's levy system was accepted by the government, and won the backing of most members of the scientific community, although there were some concerns about an absolute reign of a single funding stream and a fixed set of criteria. As an editorial in the BMJ observed, 'the pluralism of sources of research funds is much appreciated by and of benefit to research workers, who doubt that and single committee has a monopoly on wisdom' [32].

In accordance with Culyer's proposals, from 1996, the existing diverse funding streams were merged, and NHS Trusts declared net R&D costs of approximately £334 million [13]. R&D funds were then divided into two budgets. The first budget, Support Funding, was to meet infrastructure costs and fund 'own account' work. The second budget covered investigations in national priority areas, which were not funded by other non-commercial bodies [34]. In addition, a smaller, separate funding stream was made available for policy research within the Department of Health. Resources from the new fund were allocated on the basis of the previously declared costs and competitive bids [35].

The new levy was implemented amongst criticisms regarding mechanisms for its collection and distribution [13]. In 1998, the Department decided to divert £10 million of the intended levy into primary care, demonstrating that co-habitation with service delivery could be a hazard as much as a blessing for research [36]. Capacity for strategic planning within the Department, which had already been a major hurdle in operating the Rothschild system, was a further issue. An internal review of the levy in 1999 concluded that the allocation of funds needed to be based on better data collection systems, involvement of customers, and peer review of research programmes [37]. However, the review also acknowledged a clearer vision of NHS research as a consistent, evidence-based whole with a defined role in a wider scientific context.

The Culyer reforms were a major step towards putting the NHS R&D vision on a firm organisational and financial footing, against the odds of a fragmented frontline and research-averse internal market. However, the odds were resilient. In particular, it proved difficult to achieve the desired transparency of research costs and activities within organisations unaccustomed to such central scrutiny. Consultants commissioned to take stock of current activities in externally funded R&D found it difficult to find 'sufficient data ... in providers to be able to undertake a robust data collection and analysis exercise' [13,38]. Not only was change needed far beyond the R&D directorate's immediate sphere of influence. To anchor the new arrangements, the fundamental questions of research support needed to be addressed afresh: how to link up with other research support; how to set priorities; and how to ensure the equitable distribution of funds.

## A vision of needs driven excellence (2000 to 2006)

The Department of Health set about strengthening the basis of its new approach in a series of strategy papers published in 2000 and 2001 [39-41]. To achieve clear and central positioning of its investigations in the wider non-commercial research landscape, the Department promoted partnerships, the sharing of findings and interdisciplinary work through the creation of a new funders' group. Moreover, synergies with industry and the universities featured as an objective in the departmental Science Strategy.

Naturally, in the context of relationships with other research bodies, the old question of the appropriate roles of the Department, the NHS, and the MRC also needed to be answered. Here, the strategy opted for differentiation according to clinical immediacy rather than degree of direction, specifying that the MRC, along with the other Research Councils, was best placed to cater for 'research needs that are likely to require more basic input'. The NHS would cater for its needs through its national programmes, whereas the DH would commission its own research from external bodies as required. However, the legacy of blurred boundaries was difficult to overcome, and the MRC was also conceded work in areas where it commanded specific expertise [41].

If the focus on needs was to be the distinguishing feature of research in the NHS, determining these needs was of primary importance. The Department proposed to resolve this issue through the use of expert advice, stakeholder consultations, horizon scanning and systematic reviews, but did not specify how the information obtained would be weighted. Similarly, it proposed to improve priority setting through robust criteria, but did not detail how these criteria would be developed. Like the previous review committees, the authors found it easier to formulate general guiding principles than to give them content. The developments of the preceding decades had made the task no easier. Where Haldane could propagate the intuition of researchers, and Rothschild the circumscribed 'order' of the customer, the sheer range of individuals, organisations and methods now considered to have a rightful share in departmental decision-making had grown substantially. As a result, providing leadership had become a delicate balancing act, which did not lend itself to *ex ante *decisions of principle.

Finally, to clarify the allocation of funding, the Department introduced a remodelled system which differentiated between a funding stream to cover the costs of supporting R&D, and funding for work in NHS 'priorities and needs' areas [39,42]. However, the intended improvements were slow to make themselves felt at the front line. In the following period, funding levels were subject to strong fluctuations due to increases and cuts, including a further diversion of R&D funds to meet frontline delivery targets. In February 2001, the government pledged to increase the budget for NHS research and development by £30 million, bringing the total £479 m. The additional funds were earmarked for research commissioned by the Department of Health £21 million) and to boost support for NHS health care providers undertaking research (£9 million). However, in September of the same year, funds for R&D were once more re-directed, as funds for regional NHS R&D were used to meet frontline service targets [43,44].

More fundamentally, health researchers questioned the mechanism of distribution. A report by the Academy of Medical Sciences, published in 2003, argued that clinical research was receiving little of the funds 'notionally' attributed to NHS R&D. To amend the situation, the report called for funding from the MRC and the government's Office of Science and Technology to be made available for the support of clinical trials and training, and to create incentives for medical academic careers [45]. Once again, a feeling of crisis manifested itself through a foray into the territory of other public fund holders.

The Academy of Medical Sciences was not alone in questioning the functionality of the funding mechanisms. In the autumn of 2003, the Department appointed a project team to throw light on the question whether R&D funds had been used for other health service activities, as suggested by substantial local variations in the cost of services. In 2004, to counter the risk of cross-subsidies between service delivery and research, Sally Davies, Director of R&D, then called for institutions to lay open the exact use of their budgets, a basic element of Culyer's original concept for the levy. In the same year, the Treasury's Science and Innovation Investment Framework (2004) pledged to move to full transparency of the use of R&D funds allocated to NHS Trusts [46].

Recent government pledges to strengthen the fabric of the Department's strategy have also focused on funding and funding partners. In 2004, the Treasury committed to increase NHS R&D funding by £100 million by 2008. The budget also announced the creation of a new body, the UK Clinical Research Collaboration, to bring together all major stakeholders with the aim of promoting the attractiveness and competitiveness of the United Kingdom as a location for clinical research. Unlike previous bodies, this group also invited representatives from industry.

In January 2006 the history of public strategy for health research reached a new milestone in the publication of the Department of Health's new national health research strategy, 'Best Research for Best Health' [47]. The strategy represents a fresh endeavour to meet the challenges which, as we have seen, have presented themselves at every turn of the previous decades' policy development, such as investment of resources and commissioning of research.

Driven by the overall objective of harnessing research to improve the 'health and wealth' of the nation by 2010 and beyond, 'Best Research for Best Health' marks a number of significant structural changes and departures from previous practice. These include the creation of a National Institute for Health Research (NIHR) as a virtual national research facility offering attractive and effective working conditions for world class staff. From a historical perspective, the creation of this outspokenly competitive new body represents the Department's boldest step yet to exorcise the one time stigma of its researchers as 'second-class scientific citizens'. The document also introduced a new distribution model for R&D resources to replace previous trust allocations. In a bid to reconcile breath and depth of funding, the new model takes a triple approach of population-based as well as broadly and leading edge focused competitive funding.

The strategy envisions the NIHR to provide a consolidated focus for the Department's programmes of research commissioning. In the tradition of its predecessors, the document does not expand on how priorities for funding are to be determined beyond stating the aim to support 'important research which is inadequately supported by other funders', and maintaining a firm focus on identified needs. However, it highlights two key partners in establishing research programmes. The first is patients, who, from a complete absence in earlier strategies, have acceded to an (at least intentional) integral role in the 'identification, design, recruitment and dissemination of projects'. The second is the UK Clinical Research Collaboration, the forum for clinical research which comprises the MRC amongst other stakeholders. Ultimately, how funding decisions are made and implemented, and in what ways partners are involved in this process will be one of the most interesting elements of the new strategy as it is put into practice.

## Conclusion

The conditions and methods of health-related research have changed beyond recognition since the conception of the National Insurance Fund in Edwardian days. By contrast, the basic questions and tensions regarding the optimal support of such research have proved remarkably timeless. They include the balance between the control and freedom of researchers; between competing scientific fields; between the influence of scientists, policy makers and patients; and between healthcare providers' role as hosts of research and efficient players in the market.

As shown in this paper, these questions have been repeatedly debated over almost a century of publicly funded biomedical and health research in England. Some responses have been cyclical in nature, such as the degree of autonomy granted to researchers. Other questions have been answered with new models. For example, Haldane initially granted the strongest voice in setting the biomedical agenda to the researchers themselves, whereas Rothschild shifted decision making to ministry staff, and in more recent times the power has again been redistributed among a steadily growing group of stakeholders.

Finally, some issues have proved so delicate as to become unattractive for outspoken policies. This is noticeable in the areas of priority setting, and particularly regarding the balance between research that has an immediate relevance versus research of absolute quality and intrinsic future potential. In the last few decades, several committee and policy papers have circumvented this issue by neutrally presenting different arguments; others have proposed to put the decision to the vote of stakeholders. The Department's latest strategy envisages close cooperation with the UKCRC and patients, while firmly building on the premise that research can be attractive to the best minds and effective while driven by needs of the NHS.

However, the experience of the previous models described in this paper highlight the intrinsic challenges of negotiating patient need and researcher freedom, and of supporting applied research within a healthcare setting. Notably, they include generating consensus over what the most pressing needs are, coping with the competing pressures of delivery, and satisfying expectations in research performance and health outcomes. Given these challenges, no strategy, including the most recent strategy by the Department of Health, is likely to be the last word in health research policy. However, in the history of developing such approaches, 'Best Research for Best Health' is a novel departure which, more than any of its predecessors, will test the Department's potential as a leader of a world class system of national health research.

## Competing interests

This article was produced as part of research funded by the Department of Health's R&D Directorate. At the time of submission, both authors were employed by RAND Europe, an independent public policy think tank.

## Authors' contributions

MS drafted and developed the article, JG contributed to its argument and structure, and provided critical feedback on its drafts. Both authors read and approved the final manuscript.
